# Self-administered at-home lung ultrasound with remote guidance in patients without clinical training

**DOI:** 10.1186/s12931-024-02744-y

**Published:** 2024-03-05

**Authors:** Nika Elmi, Yasmin Sadri, Frank Myslik, Jordan Chenkin, William Cherniak

**Affiliations:** 1 Michael G. DeGroote School of Medicine, McMaster University, Hamilton, ON Canada; 2 Dalhousie Medical School, Dalhousie University, Halifax, NS Canada; 3 Division of Emergency Medicine, Department of Medicine, Schulich School of Medicine and Dentistry, Western University, London, ON Canada; 4 Department of Emergency Medicine, Sunnybrook Health Sciences Centre, Toronto, ON Canada; 5 Division of Emergency Medicine, University of Toronto, Toronto, ON Canada; 6 Division of Emergency Medicine, Department of Family and Community Medicine, University of Toronto, Toronto, ON Canada; 7 School of Professional Studies, Northwestern University, Chicago, IL USA; 8 Department of International Health, The Johns Hopkins Bloomberg School of Public Health, Johns Hopkins University, Baltimore, MD USA

**Keywords:** Ultrasound, POCUS, Self-imaging, Community health, Remote telemonitoring

## Abstract

**Background:**

Access to timely and accurate diagnostic imaging is essential for high-quality healthcare. Point-of-care ultrasound has been shown to be accessible and effective in many aspects of healthcare, including assessing changes in lung pathology. However, few studies have examined self-administered at-home lung ultrasound (SAAH-LUS), in particular performed by non-clinical patients (NCPs).

**Research question:**

Are NCPs able to perform SAAH-LUS using remote teleguidance and produce interpretable images?

**Study design:**

Patients were enrolled to the study in a mix of in-person and virtual recruitment, and shipped a smartphone as well as a point of care ultrasound device. Tele-guidance was provided by a remote physician using software integrated with the point of care ultrasound device, allowing real-time remote visualization and guidance of a patient scanning their own chest. A post-intervention survey was conducted to assess patient satisfaction, feasibility, and acceptability of SAAH-LUS. Two POCUS expert reviewers reviewed the scans for interpretability, and inter-rater agreement between the two reviewers was also computed.

**Results:**

Eighteen patients successfully underwent 7–14 days of daily telemedicine in parallel to daily SAAH-LUS. Across 1339 scans obtained from ten different lung zones, the average proportion of interpretability was 96% with a chance-corrected agreement, or Cohen’s kappa, reported as κ = 0.67 (significant agreement). 100% of NCPs surveyed found SAAH-LUS to be a positive experience, particularly for its ease of operation and ability to increase access to healthcare services.

**Interpretation:**

This study demonstrates that NCPs can obtain interpretable LUS images at home, highlighting the potential for SAAH-LUS to increase diagnostic capacity, particularly for rural and remote regions where complex imaging and healthcare providers are difficult to obtain.

*Trial registration* The clinical trials has been registered (clinicaltrials.gov). Registration number: NCT04967729

## Introduction

Access to timely and accurate diagnostic imaging is integral to high-quality healthcare [1]. Within the global community, 47% of the population has little to no access to diagnostics, with low and middle-income countries (LMICs) as well as under-resourced communities in high-income countries (HICs) carrying the largest burden [1]. Barriers to accessing care include the cost of diagnostic equipment, transportation of patients to healthcare facilities, and—among other factors—strained health workforce capacity [1]. The COVID-19 pandemic has clearly demonstrated the magnitude of these deficiencies, specifically, in rural, marginalized, and/or remote populations. Often, these regions have the most limited access to diagnostic equipment due to key barriers such as geographic isolation from imaging facilities and unfamiliarity with larger urban centers, where imaging is often located [2, 3]

Point-of-care ultrasound (POCUS) has helped to bridge some of these gaps by decreasing the cost and complexity of basic imaging [4]. POCUS is a safe, affordable, and simple to perform diagnostic test that has been shown to expand imaging capacity globally [5]. In LMICs, POCUS has improved health outcomes in obstetric management [6], cardiovascular diseases [7], infectious diseases [8] and even in complex humanitarian emergencies [9].

In a more focused capacity, POCUS has been shown to be an ideal modality for assessing progressive changes in lung pathology, with positive implications as a tool for self-imaging [10–14]. As well, lung ultrasound (LUS) has been recognized for its potential to reduce quantities of chest x-rays, particularly due to its increased sensitivity in the assessment of pneumonia [15]. These findings support a new generation of advanced home care and moves the needle forward on empowering patients to play a greater role in their health.

Innovative cloud-based teleradiology platforms have previously been utilized to provide asynchronous training and support to providers in low-resource settings [16]. More clinical trials, however, are needed to assess the efficacy of enabling patients with no previous training to administer LUS on themselves, at home, while paired in real-time through teleguidance with a qualified physician.

Herein, we enable self administered at-home lung ultrasound (SAAH-LUS) in non-clinical patients (NCPs) to scan their own chest for pneumonia, while paired in real-time with an Emergency Physician trained in ultrasound. The purpose of this study was to examine the ability of NCPs to produce interpretable images using SAAH-LUS, and to assess the overall satisfaction and feasibility of the experience.

## Materials and methods

### Study design

This was a prospective cohort study of a convenience sample of patients diagnosed with COVID-19.

### Inclusion criteria

Participants over the age of 18 with a nasal RT-PCR swab positive for COVID-19 and/or a COVID-19 rapid antigen test and those with access to Wi-Fi at home sufficient for video calls. Patients were non-clinical, defined as having no prior LUS training.

### Exclusion criteria

Exclusion criteria included subjects who were unwilling or unable to directly provide consent.

### Patient intake

Patients were identified either through a digital health platform’s patient intake system (Rocket Doctor Inc, RD) or through the Emergency Department (ED) at the Markham-Stouffville Hospital. Markham-Stouffville hospital is an acute care community hospital with two sites that had an average of 110,679 emergency department visits in 2022–2023 [17].

Once identified and meeting the inclusion criteria above, participants were connected with one of two study coordinators who completed an informed verbal consent.

Following consent, the study subject was connected with a logistics team member who coordinated the shipping of the ultrasound kit, as well as booking for regular telemedicine and teleguidance sessions.

### Telemedicine sessions

Patients were contacted every 2–3 days to assess their clinical condition. Metrics such as temperature, oxygen saturation, and respiratory symptoms including cough, shortness of breath, and chest pain were collected through a physician assessment. Physicians additionally documented their observations about the patients’ clinical status, any changes observed since the prior visit, and the disposition plan.

### Teleguidance sessions

**Fig. 1.**
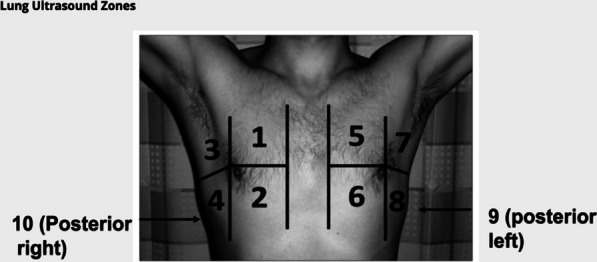
10-Zone lung protocol followed by patients during self-imaging procedures

LUS sessions every 2–3 days were conducted for 7–14 days with the patient in a semi-recumbent position. The exam included at least five points over each lung, following an established 10-zone lung ultrasound protocol (Fig. 1) [18]. Initially, 2 complete weeks with daily LUS were required as the primary outcome was to assess clinical changes in COVID-19 symptoms. However, as the study progressed, COVID-19 vaccines were rolled out and acuity of COVID-19 patients decreased. Subsequently, the ability of patients to capture satisfactory images at home, and so the interpretability of LUS images, became the primary objective of the study rather than the acuity of COVID-19.

Teleguidance sessions were supervised by an emergency physician who was certified and experienced in lung ultrasound. Image acquisition was assisted by the clinicians who could remotely adjust ultrasound pre-sets while simultaneously maintaining a live view of the patient, probe orientation and utilizing augmented reality to signal a direction of orientation of the probe to the patient.

Images were uploaded into the secure cloud storage server. The duration of each transmission with remote clinician guidance was recorded as well as a score for each image on a scale of 0–3 for severity according to the methods in Soldati et al. [19]. The Soldati scores were completed after the real-time teleguidance sessions with the patient were completed.

### Devices and training

Each patient was provided a Butterfly iQ ultrasound device (Butterfly Network, Guilford, CT) and an Apple iPhone XR (Apple Inc, Cupertino CA). Participants received a single 20-min virtual training session led by a study coordinator to learn about the operation of the ultrasound system, connection to remote guidance, and image transmission. The training occurred 1 day prior to the patient’s first teleguidance session.

### Ethics review

REB approval was obtained from the Markham-Stouffville Hospital REB, approval number 114-2005. IRB approval was obtained through Veritas IRB, approval number 2711-6311-4.

### Image review

The LUS findings were reported in a standard quality assurance (QA) document within RedCap, including image quality and interpretation [16]. One physician was responsible for collecting images with the patient in real-time while in the teleguidance session as well as providing a review on diagnostic interpretability, while a second physician over-read each scan after the visit with no knowledge of the patient or their clinical condition to collect data for inter-rate reliability. The score provided for diagnostic interpretability was not provided to the patient.

### Primary objective

The primary objective of this study was to determine whether NCPs were able to perform SAAH-LUS and obtain images that were interpretable and useful for clinical decision-making.

In order to assess this outcome an independent review by two POCUS experts was conducted. The experts met following the review to discuss discrepancies and reach a consensus. Both experts were POCUS fellowship-trained in Canada (DRCPSC, Acute Care POCUS) and have over 10 years of experience with POCUS. This utilized a modified Delphi method based off several key features from previous validated instruments [20]. as well as consensus studies using a binary assessment to define an interpretable lung ultrasound image. The modified Delphi method included a comprehensive review of reported Lung Ultrasound findings and a detailed electronic questionnaire wherein only unanimously agreed upon criteria that were deemed essential for interpretability were included. Following the initial selection round, the panel reviewed the results and reached a consensus on any discrepancies concerning criteria that members felt needed further clarification or inclusion. The physicians were blinded to the patient’s condition. The criteria required appropriate depth through visualization of at least one A-line, pleural line visualization between the ribs, appropriate gain by visualization of a hyperechoic pleural line with A-lines, and a documented recorded image of at least 4 s in length. The visibility of at least one A-line was included to ensure that the probe is over the pleura and getting an appropriate window, while also allowing the reviewer to determine if any visible B-lines would obliterate the A-lines.

### Secondary objective

The secondary objectives of this study included the overall feasibility and satisfaction by patients with performing self-administered ultrasound.

### Post-intervention survey

Following completion of the clinical trial each participant was contacted for a post-intervention survey. This was performed through a phone interview using a predefined outline of questions that assessed satisfaction, feasibility, and acceptability of the LUS using a 4-point Likert scale. Two open-ended questions were also asked as an opportunity for participants to elaborate on general feedback, and the overall experience.

### Statistical analysis

LUS scans were aggregated into the ten lung zones used for the trial across all patients. Quality assessment sheets were reviewed and interpretability assessed. Simple descriptive statistics were used using IBM SPSS software to describe the proportion of scans in each lung zone that were interpretable [21]. A paired t-test was used to compare the time required for patient’s to scan during their first and final appointments.

A kappa coefficient was calculated to test the inter-rater reliability between the expert POCUS clinicians in order to evaluate the consistency and quality of the images uploaded for determining the primary objective.

Likert scale responses to the qualitative survey were reported as a mean, and word frequency analysis was used to highlight commonly occurring words and phrases among short-answer questions.

## Results

25 patients met the inclusion criteria and were referred to the consenting team. All 25/25 (100%) participants consented to participate. Of those participants, three were subsequently unable to continue and 22/25 (88%) were shipped the lung ultrasound kit. A total of 18/25 (72%) completed the minimum of 5 telemedicine sessions. Patient attrition occurred due to various factors, such as challenges in maintaining contact (1), voluntary withdrawals (3), internet connectivity issues (including hotspot unavailability) (1), and device malfunction (1), notably a broken smartphone (1). This study was technically much more challenging as a result of launching it at the very height of the COVID-19 pandemic.

Table 1 outlines the demographics and clinical characteristics of each participant. While two registered nurses were included in the study, none had prior experience with LUS. The rest of the participants did not have any clinical background.

**Table 1:** Clinical characteristics

Average age (years)	39.4 [SD: 14.1]
Proportion male/female	40%/60%
Average BMI (kg/m^2^)	26.9 [SD: 6.6]
Average oxygen saturation	97.5% [SD: 0.6]

Table 3 outlines the number and proportion of scans that were deemed to be interpretable versus not in each lung zone, together with Soldati scores. A total of 139 completed lung studies, with approximately 10 image sets per study (1339 total scans) were included for analysis. Of the 22 patients who were initially enrolled and shipped ultrasound kits, 18 were able to begin self-scanning. For the first 6 patients we targeted daily scans for 14 days. This was found to be cumbersome for patients and clinically unnecessary, so it was adjusted to 6 scans per patient. As such, we expected a total of 156 total lung studies. We ultimately completed 139 studies.

The 18 patients who performed SAAH-LUS were contacted by phone by a research coordinator and were asked to participate in a satisfaction survey. Ultimately, 11/18 (61%) participants completed the survey. When asked about the overall experience with at-home LUS, nine short answer responses were provided. A generally positive word such as “good”, “tremendous”, “easy”, and “nice” appeared at least once in 9/9 (100%) of the free-text responses.

Participants consistently reported that the POCUS was easy to operate and felt reassurance from having immediate access to a physician. Two participants reported being happy to be able to take care of their own health. One patient wrote, “It was an exciting experience where I could take ultrasound images for myself. It was tremendous and assuring that I could have feedback directly from home about my lung health after I had covid.”

One participant highlighted needing their personal support worker (PSW) to complete the LUS due to mobility restrictions. Eleven out of 11 participants (100%) reported being likely to use the ultrasound at-home if recommended to by their physician (4 on a 4-point Likert scale). All eleven participants (100%) also indicated that they believed at-home diagnostic methods such as the LUS could save them from an in-person visit to the hospital or walk-in clinic/urgent care (4 on a 4-point Likert scale). The majority of participants also reported being satisfied with their virtual training session, the lung ultrasound sessions, and indicated that they felt, “very comfortable using the ultrasound probe by the end of the study”.

### Descriptive statistics

Overall, the average proportion of interpretable lung scans across the 1339 completed scans in ten lung zones was 96%. Across all ten lung zones, the raw agreement yielded a score of 0.98, with chance-corrected agreement, or Cohen’s kappa, reported as κ = 0.67 (significant agreement). A kappa of 0.41–0.60 was considered “moderate” agreement and 0.81–1 was considered “almost perfect” agreement [22]. Raw agreement was the ‘lay agreement’ as defined by the sum of agreement of the POCUS experts over the total sample size, without the chance of correction. The agreement statistics calculated for each lung zone is outlined in Table 2 [21]. Table 4 shows a decrease in the time to conduct the scans across all lung zones between first and final LUS appointments.

**Table 2:** Agreement statistics

Lung zone	Raw agreement	Cohen’s Kappa
1	1.0	N/A
2	0.99	0.79
3	0.97	0.48
4	0.98	0.76
5	0.99	0.89
6	0.99	0.85
7	0.99	0.49
8	0.96	0.60
9	0.99	0.74
10	0.96	0.43
All zones	0.98	0.67

## Discussion

The results from this study demonstrate the capacity for NCPs who are feeling acutely unwell to obtain interpretable ultrasound images of their own chest while paired in real-time with an expert POCUS clinician.

This study contributes to a limited but growing body of evidence recommending the validity of POCUS for self-imaging, particularly for lung ultrasound [23–28]. Our study validates its feasibility among NCPs with no prior POCUS knowledge. The results of this study show that within a short amount of time enrolled patients were able to successfully acquire diagnostic-quality LUS images for expert clinician interpretation.

These findings are applicable now, more than ever, as healthcare resources become more and more strained and diagnostic imaging limited around the world [29]. While our study encountered challenges and limitations, generally the participants felt comfortable using the device, enjoyed the experience, and would be glad to perform ultrasound on themselves from home in the future if they fall ill.

From a study-design perspective, shifting the primary outcome from COVID-19 disease progression to the feasibility of SAAH-LUS opened the doors to broadly generalizing findings to patients with any cough, cold or flu-like symptoms, or perhaps those with congestive heart failure and/or other conditions that might benefit from lung ultrasound imaging. Future studies should build on our findings by exploring its applicability among communities most limited to diagnostic services, such as those in LMICs and/or rural areas of Canada/HICs, and expanding the study outcomes to include various pathologies including but not limited to cardiovascular diseases and maternal/perinatal health.

Some technical obstacles for consideration that we encountered were difficulties in visualizing posterior lung fields (zones 9 and 10). On average, these were the most challenging for patients to obtain diagnostic-quality images (Fig. 1), although with creativity and support in some cases from family or a caregiver was easily overcome. In all study subjects, if the patient was scanned supine lung volumes would decrease, and lower lung field images would encompass subdiaphragmatic structures. In these cases, the zone of imaging had to be shifted cephalad when supine. In the sitting or standing position, much more lung parenchyma could be imaged as lung volumes increased. Barriers to image acquisition also included access to reliable and stable internet, a key issue in both Canada and around the world.

In spite of these challenges, as outlined in Table 3, 96% of all images recorded and reviewed were deemed interpretable. Cohen’s kappa was also computed across all the lung zones as an indication of interrater reliability, yielding substantial agreement between the two physicians (κ = 0.67) [22]. A further limitation was that the average reported Soldati score was low at 0.1 as the patient sample did not present with severe disease. This did not however impact the primary objective of the study, demonstrating NCPs can satisfactorily perform SAAH-LUS. Participants also showed an improvement in the time needed to scan the lung fields as they moved from first to last scan (Table 4). These trends aligned with qualitative reports of increased comfort with LUS over time. While our study focused on assessing lung ultrasound interpretability, our cohort happened to be characterized by on average a low Soldati score. We acknowledge that while the method of self-scanning remains unchanged irrespective of severity of illness, it is possible that severity may in some way change image interpretation. As such, we recommend further trials incorporate more severe lung pathologies, and their potential impact on image interpretability.

**Table 3:** Interpretability of patient images by lung zone

Lung zone	Average Soldati score	Total # of non-diagnostic reads	Total # of diagnostic reads	Percent diagnostic (%)
1	0.2	0	139	100
2	0.1	7	132	95
3	0.1	5	135	96
4	0.1	10	129	92
5	0.2	4	135	97
6	0.1	3	136	98
7	0.1	4	135	97
8	0.3	8	131	94
9	0.2	5	134	96
10	0	6	133	95
Total	0.1	52	1339	96

**Table 4:** Average time to scan across all zones

First visit	8.9 min [CI: 7.35, 10.5]
Final visit	4.2 min [CI: 3.69, 4.65]
*p-value*	0.00008

These results build on a growing body of evidence demonstrating the utility of virtual care and remote monitoring technologies in promoting patient empowerment through active and informed involvement [30]. By providing an opportunity to receive care outside of the hospital, SAAH-LUS creates a patient-centered experience that encourages autonomy and enables an empowering and accessible experience.

## Conclusions

The findings from this study support a new generation of advanced home care monitoring. It empowers patients to play a greater role in their health while also improving access to care. In a time where access to diagnostics and diagnostic services is needed most [1], SAAH-LUS could potentially help to improve quality of care and convenience for urban patients, as demonstrated in this study, while additionally providing equitable access to diagnostic imaging for patients in rural and underserved communities in HICs, as well as LMICs alike.

## Data Availability

The datasets used and/or analyzed during the current study are available from the corresponding author on reasonable request.

## Abbreviations

POCUS: Point-of-care ultrasound
LMICs: Low and middle-income countries
HICs: High-income countries
LUS: Lung ultrasound
SAAH-LUS: Self administered at-home lung ultrasound
NCPs: Non-clinical patients
